# Concomitant administration of resveratrol and resistance training ameliorates acrylamide-induced spatial learning impairment in rats

**DOI:** 10.22038/AJP.2023.22937

**Published:** 2024

**Authors:** Shaghayegh Hemat Jouy, Jafar Shahraki, Ramin Rezaee, Vahideh Ghorani, Mandana Gholami

**Affiliations:** 1 *Department of Exercise Physiology, Faculty of Physical Education and Sport Sciences, Central Tehran Branch, Islamic Azad University, Tehran, Iran*; 2 *Department of Pharmacology and Toxicology, Faculty of Pharmacy, Zabol University of Medical Sciences, Zabol, Iran*; 3 *International UNESCO Center for Health-Related Basic Sciences and Human Nutrition, Faculty of Medicine, Mashhad University of Medical Sciences, Mashhad, Iran*; 4 *Clinical Research Development Unit, Imam Reza Hospital, Faculty of Medicine, Mashhad University of Medical Sciences, Mashhad, Iran*; 5 *Applied Biomedical Research Center, Mashhad University of Medical Sciences, Mashhad, Iran*; 6 *Department of Physical Education and Sport Sciences, Faculty of Literature, Humanities and Social Sciences, Science and Research Branch, Islamic Azad University, Tehran, Iran*

**Keywords:** Acrylamide, Resveratrol, Scopolamine, Resistance training, Spatial memory

## Abstract

**Objective:**

The present study examined effects of resistance training (RT) and resveratrol (RES) alone and together on acrylamide (AC)-induced memory impairment in rats.

**Materials and Methods:**

Animals were divided into 6 groups: (1) Control group which received normal saline intraperitoneally (ip) daily for 8 weeks; (2) Scopolamine (SCO) group which received SCO (1 mg/kg/day, ip) for 8 weeks; (3) AC group which received AC (5 mg/kg/day, ip) for 8 weeks; (4) AC + RT group which received AC (5 mg/kg/day, ip) for 8 weeks and performed RT (5 days a week for 8 weeks); (5) AC + RES group which received AC (5 mg/kg/day, ip) and RES (1 mg/kg/day, ip) for 8 weeks; and (6) AC + RT + RES group which received AC (5 mg/kg/day, ip) and RES (1 mg/kg/day, ip) for 8 weeks and performed RT (5 days a week for 8 weeks). On day 53, animal training began in the Morris Water Maze (MWM) and 24 hr after the last training, the probe test was performed.

**Results:**

RT and RES alone did not significantly affect escape latency or traveled distance increased by AC. However, concomitant RES and RT treatment significantly reduced these parameters compared to the AC group. Co-treatment with RES and RT also significantly increased the time spent in the target quadrant compared to the AC group. Lipid peroxidation was reduced in the AC+RES and AC+RT+RES groups compared to the AC group.

**Conclusion:**

It seems that daily co-treatment with RES and RT for 8 weeks ameliorates the memory-impairing effects of AC.

## Introduction

Acrylamide (AC) is produced during cooking and it is found in carbohydrate-rich (150-4000 µg/kg) and protein-rich foods (5-50 µg/kg) (Tareke et al., 2002). Exposure to AC mainly occurs via consumption of high-carbohydrate diet. Other routs of exposure to AC such as transfer of AC monomers from food packaging polymers and environmental exposure through contaminated water, as well as tobacco and cosmetic products also exist (Bušová et al., 2020; Rifai and Saleh, 2020). Reproductive toxicity, carcinogenicity, muscle weakness, and neurotoxicity including memory impairment, are main toxic effects of AC (Bušová et al., 2020; Exon, 2006; Shipp et al., 2006). AC induces apoptosis in neurons and leads to hippocampal neurogenesis and learning and memory impairment (Lee et al., 2018). AC produces similar neurotoxic effects at low and high doses, however, longer duration of exposure is required at low doses (Erkekoglu and Baydar, 2014).

Resveratrol (RES) is a plant-derived polyphenolic agent with antitumor and antioxidative properties and could combat memory loss. Main food sources of RES include wine, grapes, soy and peanuts (Burns et al., 2002). RES can regulate a variety of signaling molecules such as cytokines, Wnt, caspases, matrix metalloproteinases, and nuclear factor-κB (Singh et al., 2019). RES has been reported to potentially improve treatment outcomes in human patients with metabolic syndrome, diabetes, obesity, multiple myeloma, colorectal and breast cancers, hypertension, cardiovascular disease, stroke, kidney disease, inflammatory diseases, rhinopharyngitis and Alzheimer's disease (AD) (Hashemzaei et al., 2016a; Hashemzaei et al., 2016b; Singh et al., 2019; Tabrizian et al., 2019; Tabrizian et al., 2017; Teimouri et al., 2022).

Preventive effect of RES against memory damage and its improving properties on learning have been shown (Zhang et al., 2019; Zhao et al., 2013). RES can affect neural differentiation, improve cognition and enhance hippocampal plasticity and neurogenesis. Also, RES protects dopaminergic neurons from damages involved in the pathogenesis of Parkinson’s disease (Okawara et al., 2007).

Resistance training (RT) delays memory impairment in middle-aged and healthy elderly and improves memory in the elderly (Marston et al., 2019; Norouzi et al., 2019). The useful effects of exercise on functions of the brain such as neurogenesis and neuronal synapses formation as well as its influences on mental disorders treatment, brain injuries healing, and neurodegenerative diseases prevention have been proven (Li et al., 2013). The mechanisms by which exercise affects learning and memory status are not well understood; nevertheless, these positive effects have been attributed to exercise regulatory effects on secretion of promoting neuronal growth factors, inflammatory mediators and neuronal mediators. In addition, the role of brain biogenic amines such as serotonin, norepinephrine and histamine, in useful effects of exercise on learning status has been indicated (Akhavan et al., 2008; Taati et al., 2014).

Considering chronic exposure to low levels of AC in real life, and possibility of neurological complications and memory impairment caused by exposure to this chemical and regarding the neuroprotective effects of RES and RT, in this study, the effects of 8 weeks of intraperitoneal administration of low-dose RES and 8-week resistance training, alone and concomitantly, on AC-induced spatial learning impairment in male rats were examined.

## Materials and Methods

Scopolamine (SCO) and AC were purchased from Sigma-Aldrich Chemical Co. TBA (2-thiobarbituric acid), phosphoric acid, n-butanol, KCl and tetramethoxypropane (TMP) were obtained from Merck, xylazine and ketamine from Alfasan Co., Netherlands, and RES from Pharmacognosy Department, School of Pharmacy, Mashhad University of Medical Sciences, Iran.

Male Wistar rats (200±20 g) were kept under standard conditions (12/12 hr light/dark cycle and 21±2°C) and they had free access to food and water. All experiments were performed according to the ethical principles. 

Rats were divided into the following 6 groups (n=6) (Bhawal et al., 2015; Sánchez-Aguilar et al., 2023): (1) Control group which only received normal saline intraperitoneally (ip) daily for 8 weeks; (2) SCO group which only received SCO (1 mg/kg, ip) daily for 8 weeks; (3) AC group which only received AC (5 mg/kg ip) daily for 8 weeks; (4) AC + RT group which received AC (5 mg/kg/day ip for 8 weeks) and performed RT 5 days a week; (5) AC + RES group which received AC (5 mg/kg/day ip for 8 weeks) and RES (1 mg/kg/day ip) for 8 weeks and (6) AC + RT+ RES group which received AC (5 mg/kg/day ip for 8 weeks) and RES (1 mg/kg/day ip) for 8 weeks and performed RT 5 days a week. 

During the 8 weeks, rats of group 4 and 6 performed RT 5 days a week, using a 1-m ladder with 2-cm grid steps inclined at 85-degree angle (Lee et al., 2004). 

SCO is a muscarinic anti-cholinergic agent and can disrupt memory performance and learning. The dose of SCO in this study (1 mg/kg/day) was chosen based on previous studies that have proven its memory-destroying effects (Bejar et al., 1999; Bhuvanendran et al., 2018). 

It was shown that high-dose (50 mg/kg) AC induces memory impairment. But *in vivo* exposure to low-dose AC daily for 4 weeks (2, 20, or 200 μg/kg) exerted no apparent marked effect on hippocampal neurogenesis (Lee et al., 2018). Also, AC at a dose of 10 mg/kg/day for 7 weeks could cause memory impairment in rats (Prasad 2014). Based on the above studies, 5 mg/kg/day was selected as suitable dose for memory-destructive effects of AC.

The results of a study showed that 4-week treatment with RES (10 mg/kg/day) prevents the memory impairment caused by doxorubicin (Moretti et al., 2021). The lowest effective dose of RES in previous studies was 1 mg/kg/day (Carter et al., 2014). The reason for using low-dose RES (1 mg/kg/day) in the present study was to examine whether RES exhibits neuroprotective effects at levels close to those achievable in the diet.

**Figure 1:**
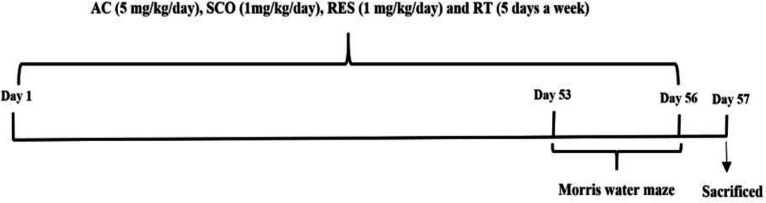
A schematic presentation of the interventions and study design

On day 53 of the study, animal training in the Morris water maze (MWM) began and continued for 4 days. The animals were transferred to the MWM (24 hr after the last training) to evaluate the stabilization of the acquired spatial memory and they underwent the probe test. Then, the rats were anesthetized using ketamine (60 mg/kg, i.p) and xylazine (6 mg/kg i.p) and decapitated; next, the hippocampus was homogenized for malondialdehyde (MDA) level determination (Hosseinzadeh et al., 2007). A schematic time-course of interventions and study design is presented in Figure 1. 

**Morris water maze (MWM) test**

Learning was evaluated in the MWM. The MWM includes a pool divided into 4 quadrants filled with water (25 cm deep, 22±2ºC) and equipped to a hidden platform. The animals received 4 acquisition trials per day for 4 days. In each trial, a rat was allowed to swim for 60 sec to find the hidden platform. If rats did not find the platform within the 60 sec time period, they were directed on the platform and remain there for 30 sec. The behavior of the animal in the MWM was assessed by the EthoVision Pro program. The latency to find the platform, traveled distance and velocity were recorded. On the 5^th^ day, the platform was removed and a probe trail was performed to evaluate spatial memory. In probe trail, animals were allowed to swim for 60 sec. The time spent in the target quadrant (where the platform was located) was compared among the groups (Tabrizian et al., 2019). 

**Measurement of lipid peroxidation**

MDA concentration as a useful biomarker of lipid peroxidation was determined in the hippocampus according to the protocol explained previously (Hosseinzadeh et al., 2007) . In this method, reaction of MDA and thiobarbituric acid (TBA) produces a red color complex that has peak absorbance at 532 nm.

**Statistical analysis**

Statistical analysis was done by SPSS 20 software. All data was first checked for normality by the Kolmogorov-Smirnov test. The data were compared among experimental groups using One-way ANOVA and Tukey’s *post hoc*. A p<0.05 was considered significant.

## Results

**Comparison of the effects of AC and SCO on spatial memory acquisition impairment assessed by MWM**

**Figure 2:**
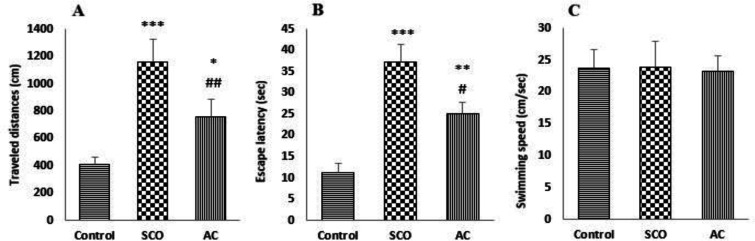
Comparison of the effect of acrylamide (AC) and scopolamine (SCO) on MWM parameters; traveled distance (A), escape latency (B) and swimming speed (C) in male rats. Values are expressed as Mean±SD (n=6). One-way analysis of variance and Tukey–Kramer *post hoc* test were used for data analysis. *p<0.05, **p<0.01 and ***p<0.001 indicate significant differences as compared with the control group. #p<0.05 and ## p<0.01 indicate significant difference as compared with the scopolamine group.

The comparison of the effects of AC and SCO on spatial memory acquisition learning relative to the control group showed that both SCO and AC significantly increased traveled distance and escape latency (Figure 2). However, no significant differences were found among the control, SCO and AC groups in swimming speed. OF note, SCO significantly increased traveled distances and escape latency compared to the AC group (Figure 2). 

**Effects of RES and RT on AC-induced spatial memory acquisition in MWM**

**Figure 3:**
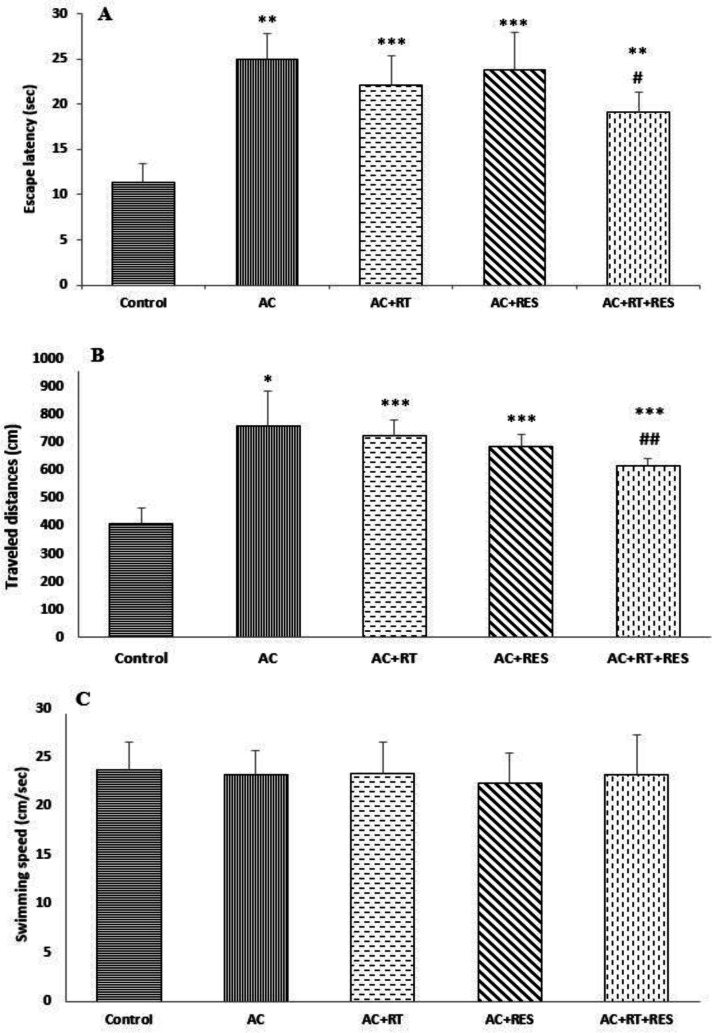
Effects of resveratrol (RES) and resistance training (RT) on MWM parameters; escape latency (A), traveled distance (B) and swimming speed (C) in male rats. Values are expressed as Mean±SD (n=6). One-way analysis of variance and Tukey–Kramer *post hoc* test were used for data analysis. *p<0.05, **p<0.01 and ***p<0.001 indicate significant differences compared with the control group. #p<0.05 and ##p<0.01 indicate significant differences compared with the acrylamide (AC) group.

The results showed that AC could increase traveled distance and escape latency. We found that RT and RES alone could not significantly reduce escape latency or traveled distance caused by AC, but their concomitant administration significantly decreased traveled distance and escape latency. Escape latency and traveled distance were significantly different in RT and RES alone treatments as well as their concomitant treatment relative to control group (Figure 3).

However, there was no significant difference in swimming speed among the studied groups (Figure 3).

**Probe test findings**

**Figure 4:**
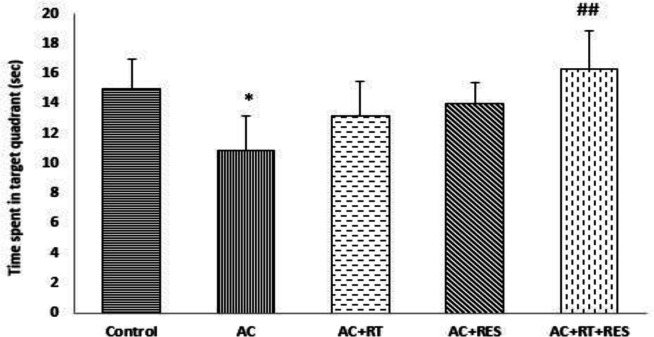
Effects of resveratrol (RES) and resistance training (RT) on acrylamide (AC)-induced spatial memory impairment in Probe test in MWM in male rats. Values are expressed as Mean±SD (n=6). One-way analysis of variance and Tukey–Kramer *post hoc* test were used for data analysis. *p<0.05 shows a significant difference compared with the control group. ##p<0.01 shows a significant difference compared with the acrylamide group.

We found that AC led to a significant reduction in the time spent in the target quadrant (i.e. where the hidden platform was located on the training days) compared to the control group. Also, the results showed that RT or RES alone could not significantly decrease the time spent in the target quadrant relative to the control group. But, simultaneous administration of RES and RT significantly elevated the time spent in the target quadrant compared to the AC group (Figure 4).

**Effect of RES and RT on lipid peroxidation caused by AC**

**Figure 5:**
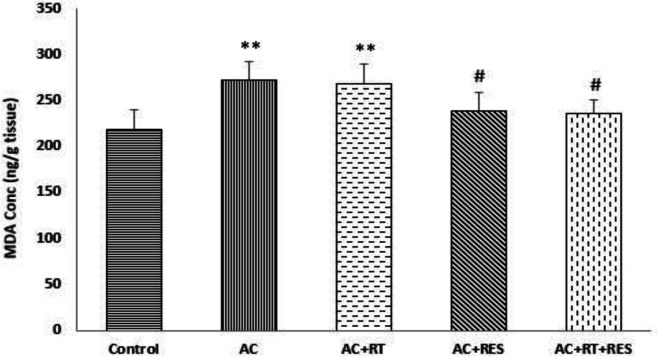
Effects of resveratrol (RES) and resistance training (RT) on lipid peroxidation. Values are expressed as Mean±SD (n=6). One-way analysis of variance and Tukey–Kramer *post hoc* test were used for data analysis. **p<0.01 shows a significant difference compared with the control group. # p<0.05 shows a significant difference compared with the acrylamide (AC) group.

Our data revealed that AC increased lipid peroxidation compared to the control group. RT could not prevent the lipid peroxidation induced by AC, whereas RES was able to significantly reduce it. Also, concomitant RT and RES administration significantly ameliorated AC-induced lipid peroxidation. A statistically significant difference was also found between RT treatment and control group in lipid peroxidation (Figure 5). 

## Discussion

This experimental study was conducted to assess the effects of concomitant administration of RES and RT on acrylamide (AC)-induced spatial learning impairment in rats. 

In the present study, intraperitoneal administration of AC for 8 weeks similar to SCO caused significant spatial learning impairment as reflected by increases in distance traveled and escapes latency, as well as decreases in the time spent in the target quadrant. Cholinergic neurons play a key role in learning and memory function (Basaure et al., 2019). SCO is an acetylcholine receptor antagonist that deteriorates memory function by inhibiting cholinergic neurons (Basaure et al., 2019). But, the cholinergic pathway is not the only contributor to memory degradation, and substances that affect dopaminergic, GABAergic, serotonergic, histaminergic, adrenergic, glutaminergic, or opioid systems can cause memory impairment through various other mechanisms (Izquierdo et al., 1992). AC is a chemical compound that has neurotoxic effects (Wang et al., 2021). AC can attach sulfhydryl groups on cysteine residues proteins and provoke the destruction of neuronal terminals and inhibition of axonal transmission (Xu et al., 2014). Also, by activating glial cells, reducing the binding protein of the phosphorylated cAMP response factor (p-CREB) and accumulation of hyperphosphorylated tau protein in the hippocampus and cerebral cortex, it impairs spatial memory (Yan et al., 2018). Most studies reporting AC-induced acute neurotoxicity in animal have examined AC doses around 50 mg/kg/day (Motamedshariaty et al., 2014; Prasad, 2014). In a rat model, memory impairment was caused by AC at a dose of 10 mg/kg/day for 7 weeks (Prasad, 2014). 

Studies have proven the useful effects of RES on memory and learning (Karalis et al., 2011; Schmatz et al., 2009; Yazir et al., 2015). Karalis et al. reported that RES (90 mg/kg) improves behavioral deficits and brain injury in the neonatal rats (Karalis et al., 2011). In stressed rats, administration 5 or 20 mg/kg RES for 35 days ameliorated emotional learning and spatial memory (Yazir et al., 2015). In a diabetic rat model induced by streptozotocin, treatment of animals with RES 10 or 20 mg/kg/day also prevented memory deficits (Schmatz et al., 2009). 

Acetylcholinesterase (AchE) inhibitors are useful treatments against AD and RES as an AchE inhibitor may probably prevent memory loss (Schmatz et al., 2009). Another neuroprotective mechanism of RES in prevention of neurodegenerative diseases is through the extracellular signal-regulated kinases (ERKs) pathway since RES is regarded as an inducer of ERK activity    (Maher et al., 2011; Tabrizian et al., 2017). In addition, β-secretase plays a key role in initiating the production of beta amyloid plaques (Broadwell and Sofroniew, 1993) and its activity is increased in AD (Citron, 2004). RES can also inhibit β-secretase enzyme and suppress amyloid plaques production, thereby preventing AD (Seino et al., 2018).

Although evidence supports that RES has benefits for memory and learning; our results showed that RES administration alone did not have any significant effect on preventing AC-induced spatial memory impairment. It seems that the low dose of RES used in the present study could not produce a significant effect when applied alone. The reason for using low-dose RES in the present study was to examine whether RES exhibits neuroprotective effects at levels close to those achievable in the diet.

On the other, numerous studies have confirmed the effect of exercise on physical and mental health. It was reported that exercise could be useful for brain-related functions such as neurogenesis and neuronal synapses, improvement of mental disorders and brain injuries, and inhibition of neurodegenerative diseases (Li et al., 2013). While neuropsychological effects of aerobic exercise are well known, our information on the protective effects of RT is still not enough. Exercise not only improves learning and memory, but also protects against brain resorption and memory disorders caused by diseases such as AD, and RT reduced beta-amyloid plaques in an AD model in a different way from aerobic exercise (Schimidt et al., 2019). RT causes delay in memory impairment in healthy middle-aged and elderly people (Marston et al., 2019). In addition, RT could enhance memory in the elderly (Norouzi et al., 2019). Spatial memory using aerobic and RT is improved through different molecular mechanisms, and RT seems to be as effective as aerobic exercise in strengthening spatial memory. RT can enhance memory associated with the hippocampus, along with increasing insulin-like growth factor-1 (IGF-1) (Cassilhas et al., 2012), increasing CREB (Suijo et al., 2013) or inhibiting β-secretase and reducing the production of amyloid plaques (Jang and Koo, 2020). 

Despite the documented evidence on RT improving brain function and preventing memory impairment, in the present study, RT alone had no significant effect on prevention of AC-caused learning impairment.

The hippocampus is a brain region with a major role in learning and memory (Anand and Dhikav, 2012). Based on the evidence, hippocampal cells are more sensitive to biochemical changes than other areas of the brain. Within the hippocampus, pyramidal and granule cells in dentate gyrus–cornu ammonis (CA)3 system are susceptible areas to oxidative stress. Therefore, oxidative damage of hippocampus can affect learning and memory through disturbing dendritic structures, neurogenesis, normal synaptic neurotransmission, etc. (McEwen, 2008; Salim, 2017; Wood et al., 2010). In the present study, lipid peroxidation in the hippocampal tissue was evaluated. The results showed that while AC increased lipid peroxidation compared to the control, RES significantly reduced it. AC was shown to induce ROS overproduction, lipid peroxidation and apoptosis (Deng et al., 2021; Yang et al., 2021). ROS are normally produced in the cell and are neutralized by the cellular antioxidant defense system. Oxidative stress refers to a state where the amount of ROS production exceeds the neutralization capacity of the cellular antioxidant defense system (Hashemzaei et al., 2020). ROS act as essential signaling agents in cognition, learning and memory formation and play critical roles in aging and the occurrence of neurodegenerative diseases such as AD (Kishida and Klann, 2007) . RES exerts free radical scavenging ability, and antioxidant properties, and it has the potential to combat lipid peroxidation and boost the cell antioxidant activity (Gu et al., 2021). It has indicated that RES, via suppression of ROS levels in the brain, could improve cognitive impairment in rats (Lin et al., 2018). Assaran et al. reported that ellagic acid, a polyphenolic compound similar to RES, reduced SCO-induced memory impairment by reducing oxidative stress (Assaran et al., 2022). 

Additionally, Cui et al. also showed that treadmill exercise for 8-week alleviated learning and memory via inhibition of oxidative stress in a spatial memory impairment rat model induced by ovariectomy (Cui et al., 2017).

Finally, the findings suggest that concurrent administration of RES and RT can ameliorate AC-induced memory impairment. This improvement was reflected by reduction in distance traveled and escapes latency, but increases in the time spent in the target quadrant, as well as decrease in MDA level in the hippocampus. In this study, no difference was observed in swimming speed among the different groups. Swimming speed is a proper measure of an animal's ability to move and lack of statistical difference in this criterion among the groups indicates that the motor function of the animals was not affected (Sharifzadeh et al., 2005).

Of note, since RT and RES through various mechanisms such as increasing CREB or inhibiting β-secretase and reducing the production of amyloid plaques can prevent AC-induced memory impairment, mechanistic investigations are recommended.

The results of the current study showed that intraperitoneal injection of AC impaired spatial learning and memory as reflected by increases in distance traveled and escapes latency, as well as decreases in the time spent in the target quadrant. In concomitant treatment, RES along with RT prevented the AC-induced impairment of spatial memory in the MWM and attenuated hippocampal lipid peroxidation. Future studies are recommended to reveal the underlying mechanisms.
